# Lesions of Acetylcholine Neurons in Refractory Epilepsy

**DOI:** 10.5402/2012/404263

**Published:** 2012-08-09

**Authors:** Masaharu Hayashi, Keisuke Nakajima, Rie Miyata, Naoyuki Tanuma, Tohru Kodama

**Affiliations:** ^1^Department of Brain Development and Neural Regeneration, Tokyo Metropolitan Institute of Medical Science, Tokyo 156-8506, Japan; ^2^Department of Psychophysiology, Tokyo Metropolitan Institute of Medical Science, Tokyo 156-8506, Japan

## Abstract

We have examined brainstem lesions in patients with refractory epilepsy disorders, including West syndrome (WS), Lennox-Gastaut syndrome (LGS), and dentatorubral-pallidoluysian atrophy (DRPLA). Acetylcholinergic neurons (AchNs) in the pedunculopontine tegmental nucleus (PPN) are involved in mental development, and disruption of neuronal nicotinic acetylcholine receptors can lead to epilepsy. In order to investigate the involvement of lesions of AchNs in refractory epilepsy, we performed immunohistochemical analyses of AchNs in the PPN in autopsy cases who had a past history of WS and/or LGS and in DRPLA cases who showed progressive myoclonic epilepsy. In addition, we performed a preliminary quantification of the levels of acetylcholine, neuropeptides, and monoamine metabolites in the cerebrospinal fluid (CSF) of patients with WS and benign convulsions associated with mild gastroenteritis (CwG). In the PPN analysis, the total number of neurons and the number of AchNs were reduced in WS/LGS and WS cases, while DRPLA cases showed a decrease in the number and percentage of AchNs. In the CSF analysis, WS patients demonstrated a reduction in the levels of inhibitory neuropeptides, while CwG patients showed increased levels of acetylcholine and decreased levels of serotonin metabolites. These data suggest the possible involvement of lesions of AchNs in WS and DRPLA.

## 1. Introduction

West syndrome (WS), which consists of tonic spasms, psychomotor developmental delay, and characteristic electroencephalography changes involving hypsarrhythmia, can have various causes, including congenital brain anomalies and sequelae after perinatal hypoxic and ischemic encephalopathy (HIE) [1]. Lennox-Gastaut syndrome (LGS) usually develops subsequently in patients with symptomatic WS. Although many anticonvulsants have been developed and treatments with adrenocorticotropins and ketogenic diets have been elaborated, epileptic seizures tend to be resistant to anticonvulsant therapies, and the mental prognosis remains poor in most patients with WS and/or LGS [2]. Progressive myoclonus epilepsy (PME) is another entity of refractory epilepsy, and the etiologies of PME include various disorders, such as neuronal ceroid-lipofuscinosis and Unverricht-Lundborg disease [3]. Dentatorubral-pallidoluysian atrophy (DRPLA), which is an autosomal dominant neurodegenerative disorder, is one of the representative diseases that cause PME in Japan [4]. DRPLA is classified into 3 major types: (1) juvenile DRPLA with onset before the age of 20 and PME, (2) early adult type with onset between 20 and 40 years of age, ataxia, choreoathetosis, dementia, and PME, and (3) late adult type with a diagnosis made after 40 years of age often developing ataxia and dementia but not PME [5]. PME is refractory to anticonvulsant therapies in patients with DRPLA, and almost all patients have poor seizure and mental prognoses [4]. In order to clarify the epileptogenesis and contribute to the development of new treatments of refractory epilepsy, we have examined lesions of monoaminergic neurons in the brainstem of autopsy cases of WS/LGS and DRPLA [6, 7]. 

Acetylcholinergic neurons (AchNs) in the nucleus basalis of Meynert (NbM) and the pedunculopontine tegmental nucleus (PPN) are involved in mental development and learning abilities [8]. Lesions of the AchN system have been observed in developmental brain disorders, such as Down syndrome and Rett syndrome [9]. The PPN, which is in the lower midbrain, contains AchNs, catecholaminergic neurons (CANs), GABAergic interneurons (GABAis), and nonacetylcholinergic and noncatecholaminergic neurons and has afferent and efferent connections to the cerebrum and spinal cord [10]. The cholinergic innervation from the PPN to the thalamus and pons is involved in the generation of muscle tone and rapid eye movement sleep, and the PPN is believed to be a part of the mesencephalic locomotor region. In controls, there is an age-dependent change in the percentages of AchNs and CANs in the PPN. The percentages of AchNs and CANs are 20% to 30% and 10% to 15% in the PPN in children and young adults, respectively, while the percentage of AchNs decreases to 5% to 10% and that of CANs increases to 20% to 30% in middle-aged and elderly subjects [11]. We have performed an immunohistochemical analysis and clarified the selective lesioning of AchNs in the PPN and/or the NbM in patients with perinatal disorders [11], Prader-Willi syndrome [12], and xeroderma pigmentosum [13], which is caused by inherited disturbances in nucleotide excision repair. However, neuronal nicotinic acetylcholinergic receptors (nAChRs), which mediate cholinergic signaling, are involved in the pathogenesis of a number of neurological disorders [14]. The identification of mutations in the neuronal *α*4*β*2 nAChRs is well known to be a key for the understanding of the pathogenesis of autosomal dominant nocturnal frontal lobe epilepsy (ADNFLE) [15]. Nevertheless, lesions of AchNs have never been examined in either autopsy brains or the cerebrospinal fluid (CSF) of patients suffering from refractory epilepsy.

In order to investigate the involvement of the acetylcholinergic neuronal system in epileptogenesis, we performed an immunohistochemical analysis of AchNs, CANs, and GABAis in the PPN in autopsy cases with a history of WS and/or LGS and DRPLA. In addition, we preliminarily evaluated the CSF levels of acetylcholine, neuropeptides, and monoamine metabolites in patients with WS and benign convulsions associated with mild gastroenteritis (CwG) (disease controls).

## 2. Materials and Methods

### 2.1. Subjects in the Pathological Analysis

 The clinical subjects included 6 cases with a history of both WS and LGS (WS/LGS cases), 6 cases with a history of only WS (WS cases), and 7 cases with clinically and genetically confirmed juvenile and early adult types of DRPLA showing PME in addition to 6 controls with no pathological changes in the central nervous system; subjects were aged from 4 months to 40 years (Table 1). Congenital brain malformation was the etiology in 4 of 6 WS/LGS cases and in 5 of 6 WS cases. The ethical committee of the Tokyo Metropolitan Institute of Medical Science approved this study, and the family of each subject provided informed consent for the postmortem analysis.

### 2.2. Immunohistochemistry

 Brains were fixed in a buffered formalin solution. Each formalin-fixed brain was cut coronally and then embedded in paraffin. Six *μ*m thick serial sections were cut from the lower midbrain, including the PPN. After microwave antigen retrieval, each section was treated with mouse monoclonal antibodies to microtubule-associated protein 2 (MAP2; 1 : 100, Upstate Cell Signaling Solutions, Billerica, MA, USA), acetylcholinesterase (AchE; Affinity Bioreagents, Inc, Golden, CO, USA), tyrosine hydroxylase (TH; Affinity Bioreagents, Inc), calbindin-D28K (CD; Novocastra Laboratories, Newcastle upon Tyne, UK), and glial fibrillary acidic protein (GFAP; Nichirei Corporation, Tokyo, Japan) at the following concentrations: 1 : 1 (GFAP), 1 : 100 (MAP2 and CD), 1 : 250 (AchE), and 1 : 400 (TH). Antibody binding was visualized using the avidin-biotin-immunoperoxidase complex method (Nichirei Corporation) according to the manufacturer's protocol. No staining was detected in the sections in the absence of antibody.

### 2.3. Quantitative Evaluation in the PPN and Data Analysis

The PPN was identified dorsolateral to the rostral superior cerebellar peduncle and the medial lemniscus in the lower midbrain, as previously reported [11]. The PPN is composed of clusters of moderately large neurons (pars compacta) and the more widespread pars dissipata in the rostral and medial regions. In the pars compacta of the PPN, the number of neurons that were immunoreactive for MAP2, AchE, TH, and CD was determined after the manual labeling of appropriate neurons with nucleoli in 2 serial sections, and the mean value was calculated. The percentages of neurons that were immunoreactive for AchE, TH, and CD relative to those that were immunoreactive for MAP2 were also calculated. All results are presented as the mean (SD). Kruskal-Wallis tests were used to analyze the differences in the data among controls and cases of WS/LGS and WS for a quantitative evaluation of immunoreactive cells. Comparisons of data between DRPLA cases and controls were analyzed by nonparametric Mann-Whitney *U* tests. 

### 2.4. Subjects in the Analysis of the CSF

 Clinical subjects included 3 infants with clinically and electrophysiologically confirmed WS, 5 patients with CwG, and 5 normal controls with acute viral infections who lacked neurological abnormalities at the follow-up examination; subjects were aged from 1 month to 2 years (Table 2). CwG is a situation-related seizure disorder that is characterized by an age at onset of 1 to 3 years, clustering seizures, normal interictal electroencephalography and neuroimaging, and favorable outcomes, as previously reported in Japan [16]. The CSF samples were obtained from 8 epileptic patients before the start of anticonvulsant therapy and from 5 normal controls at the time of examinations of fever. They were immediately frozen and stored in a −80°C freezer until the measurement. The ethical committee of the Tokyo Metropolitan Institute of Medical Science approved this analysis, and the family of each subject provided informed consent for the utilization of CSF samples.

### 2.5. Quantitative Evaluations of Acetylcholine, Neuropeptides, and Monoamine Metabolites in the CSF and Data Analysis

Concentrations of acetylcholine, neuropeptides, including aspartate, glutamate, glycine, and GABA, and monoamine metabolites, such as 3-methoxy-4-hydroxyphenylethylglycol (MHPG), homovanillic acid (HVA), and 5-hydroxyindoleacetic acid (5-HIAA), in the CSF were determined with high performance liquid chromatography (ECD-300, Eicom Corporation, Kyoto, Japan). All results are presented as the mean (SD), and Kruskal-Wallis tests were used to analyze the differences in the data among controls and patients with WS and CwG. 

## 3. Results

### 3.1. Pathological Analysis in the PPN

**Figure 1:**
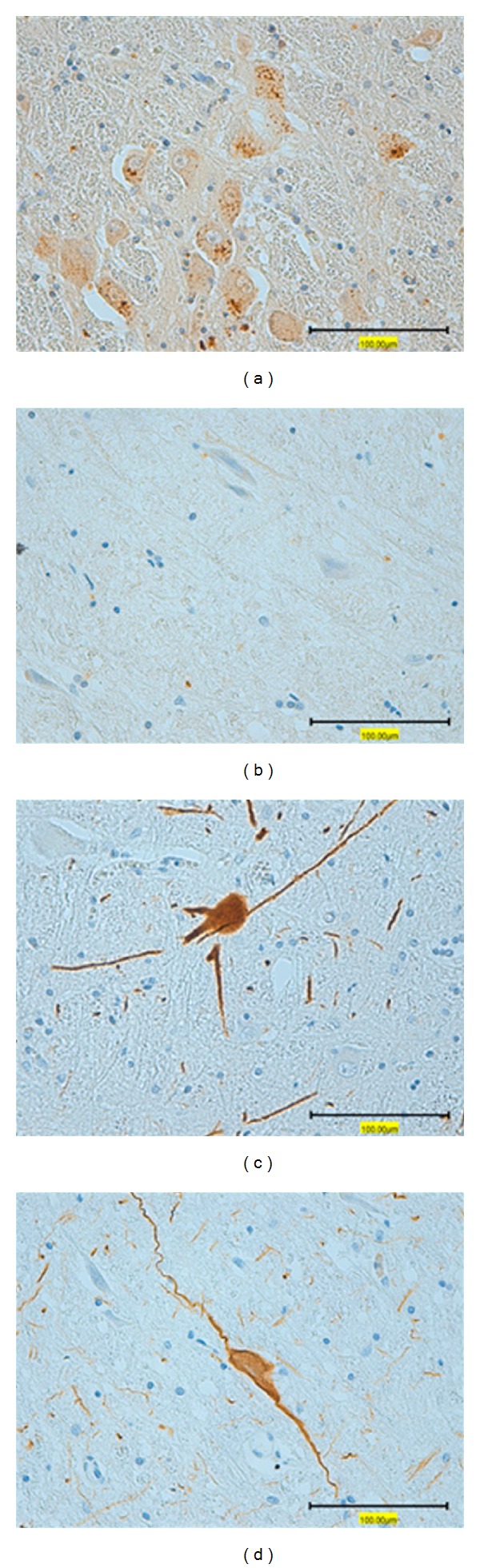
Representative illustrations of immunohistochemistry in the pedunculopontine tegmental nucleus. Photographs (a) and (c) are from a 22-year-old control who died of disseminated intravascular coagulation syndrome. Photographs (b) and (d) are from a 21-year-old case of lissencephaly with a history of West syndrome and Lennox-Gastaut syndrome. In the immunohistochemistry for acetylcholinesterase, the control showed many immunoreactive neurons (a), which were reduced in case (b). In the immunohistochemistry for tyrosine hydroxylase, immunoreactive neurons with neuronal processes were identified commonly in the control (c) and the case (d). Bars = 100 *μ*m.

**Figure 2:**
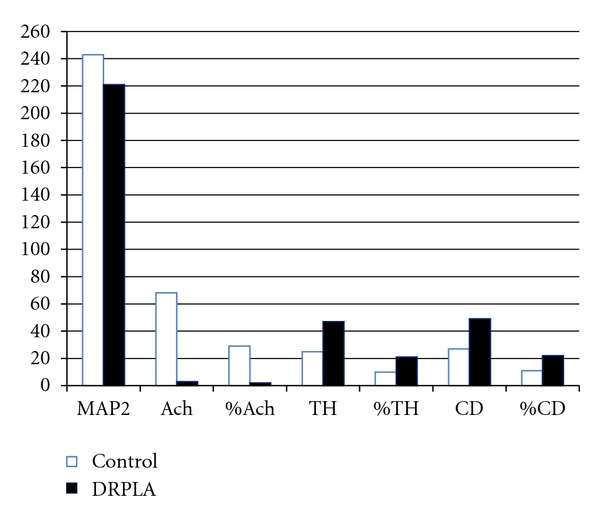
Data of the quantitative analysis in the pedunculopontine tegmental nucleus of cases with dentatorubral-pallidoluysian atrophy (DRPLA). Open and closed columns denote the numbers and percentages of each marker in controls and DRPLA cases, respectively. M: months; y: years; MAP2: microtubule-associated protein 2; AchE: acetylcholinesterase; TH: tyrosine hydroxylase; CD: calbindin-D28K.

The numbers of MAP2-immunoreactive neurons and AchNs immunoreactive for AchE were reduced significantly in WS/LGS and WS cases (Table 3 and Figures 1(a) and 1(b)). The percentage of AchNs in those cases seemed to be fewer than that of controls, but the difference was not significant. There was no relationship between the etiology and the reduction of neurons (data not shown). In contrast, there were no significant differences between controls and WS cases in the number and percentages of either CANs or GABAi, which were immunoreactive for TH or CD, respectively (Table 3 and Figures 1(c) and 1(d)). DRPLA cases showed a significant reduction in the number and percentage of AchNs (*P* < 0.01), although there was no significant difference in the number of MAP2-immunoreactive neurons. There were no changes in the numbers or percentages of either CANs or GABAi (Figure 2). There were no increases in GFAP-immunoreactive astrocytes in WS/LGS, WS, or DRPLA cases (data not shown).

### 3.2. CSF Analysis

 WS patients demonstrated a significant reduction in the levels of inhibitory neuropeptides (glycine and GABA) but not of those of acetylcholine, excitatory amino acids, or monoamine metabolites (Table 4). Such reductions in the CSF levels of inhibitory neuropeptides had no relationship with either disease type or prognosis (data not shown). In contrast, CwG patients showed a significant increase in the CSF levels of acetylcholine and a significant decrease in the CSF levels of 5-HIAA, which is a serotonin metabolite, whereas there were no changes in those of either amino acids or CAN metabolites (Table 4).

## 4. Discussion

Mutations in the neuronal nAChR subunit genes *CHRNA4*, *CHRNB2*, and probably *CHRNA2* have been demonstrated to cause ADNFLE [17]. Inasmuch as neuronal nAChRs participate in synaptic plasticity and are involved in learning, memory, and development, disruptions or alterations of nicotinic cholinergic mechanisms lead to nervous diseases, including Alzheimer's disease, autism, and epilepsy [18]. The thalamus and the cortex are innervated by cholinergic neurons projecting from the brainstem, and an imbalance between excitation and inhibition in the mutation of nAChRs may generate epileptic seizures by disturbing the thalamocortical circuits [19]. Of interest, lamotrigine, which is an anticonvulsive drug that is effective for the treatment of various epileptic syndromes, has been shown to block *α*4*β*2 nAChRs-mediated currents but to not affect glutamate- or GABA-induced currents in rat dopaminergic neurons [20]. These findings strongly indicate the significance of lesions of AchNs in epileptogenesis. The morphological identification of the neuronal nAChR remains to be elaborated in autopsy specimens, and we performed an immunohistochemical analysis on the lesions of AchNs themselves in the brains, in addition to the preliminary determination of the levels of acetylcholine, neuropeptides, and monoamine metabolites in the CSF.

The cases of WS/LGS and WS had a reduction of AchNs in addition to decreases in total neurons in the PPN (Table 3). However, the DRPLA cases showed a reduction in the number and percentage of AchNs in the preservation of total neurons (Figure 2). Similarly, in our previous immunohistochemical study on CANs in the brainstem, WS/LGS cases demonstrated a reduction in the levels of expression of TH and tryptophan hydroxylase [6], whereas such expression was preserved comparatively well in DRPLA cases [7]. It is likely that the differences in the lesions of AchNs and CANs in the brainstem may be related to the generation of specific phenotypes of convulsions in each epileptic syndrome. Intriguingly, the absence of gliosis and the comparative preservation of CANs and GABAis were commonly observed in the PPN in WS/LGS, WS, and DRPLA cases. In contrast, cases of perinatal brain damage showed a reduction in the percentage of AchNs and a compensatory increase in the percentage of CANs in addition to gliosis [11]. It is well known that cases with a history of WS and DRPLA cases exhibited small tegmentums, especially in the pons [21, 22]. These findings indicate that the reduction of AchNs is a specific change and may not be caused by destructive lesioning of the PPN itself. Some authors have speculated that WS may result from temporal desynchronization of several CNS developmental processes [23]. Accumulation of mutant DRPLA protein also involves a wide range of CNS regions far beyond the systems previously reported to be affected [24]. Because the PPN has afferent and efferent connections to both cortical and subcortical structures, selective lesioning of AchNs is likely to be involved in epileptogenesis in WS, LGS, and DRPLA. 

The Prader-Willi syndrome case showed a reduction of AchNs in the PPN but not in the NbM [12], whereas cases of xeroderma pigmentosum group A had such reductions in both nuclei [13], suggesting the presence of different regional patterns in the impairment of AchNs. Although Tsuchiya et al. reported the absence of significant neuronal loss in the NbM in DRPLA cases [25], further immunohistochemical evaluations are needed in the NbM in cases of WS, LGS, and DRPLA.

The CSF levels of neurotransmitters, neuropeptides, and their metabolites have been examined in patients with refractory epilepsy, for example, WS patients tend to show changes that are related to an impairment of serotonergic neurons and GABAis [26], and DRPLA patients demonstrate an age-related increase of amine precursors (tryptophan and tyrosine) [27]. However, the results are heterogeneous and variable, which is probably due to differences in etiology, analysis systems, and the selection of control samples. More recent and elaborate analyses in patients with early-onset severe epileptic encephalopathy failed to identify changes in the CSF levels of monoamine metabolites [28]. The CSF levels of acetylcholine were reported to be reduced in patients with PME, although that has rarely been examined [29]. 

In our preliminary CSF analysis, we examined 3 WS infants just before treatment and CwG children as disease controls, but not patients with DRPLA, in which the CSF samples were not acquired. It is interesting that 3 WS patients demonstrated changes in the CSF levels of glycine and GABA, whereas there were no significant differences in those of excitatory neuropeptides, acetylcholine, and monoamine metabolites (Table 4). The reduced levels of GABA seem to be in accordance with the findings in a previous report [26]. In order to obtain a definite conclusion, it is required to increase the number of patients and pay careful consideration to differences in etiology and prognosis. However, it is surprising that 5 CwG patients showed a significant change in the CSF levels of acetylcholine and serotonin metabolites (Table 4). Generally, seizure and developmental prognosis is good in patients with CwG, but the detailed pathogenesis has not been clarified yet [16, 30]. It is possible that transient alterations of neurotransmitter metabolism are involved in CwG.

In conclusion, the pathological study and preliminary CSF analysis suggested the possible involvement of lesions of AchNs in the pathogenesis of WS/LGS, DRPLA, and CwG. The selective reduction of AchNs was verified in the PPN in WS and DRPLA. In order to reach a definite conclusion, we should perform similar immunohistochemical examinations in cases with PME other than DRPLA and should expand the number of patients in the CSF analysis.

## Figures and Tables

**Table 1:** Summary of the subjects in the pathological analysis.

Age/sex	Cause of death
Control	
4 m/male	Peritonitis
1 y/male	Malignant lymphoma
6 y/male	Acute leukemia
22 y/male	DIC
29 y/male	Migraine
38 y/female	Heart failure

	Etiology

West syndrome/Lennox-Gastaut syndrome	
5 y/female	Lissencephaly
8 y/male	Lissencephaly
10 y/female	Lissencephaly
13 y/male	Perinatal HIE
18 y/male	Perinatal HIE
21 y/male	Lissencephaly
West syndrome only	
5 y/female	Microdysgenesis
13 y/female	Microdysgenesis
16 y/male	Heterotopia
20 y/male	Perinatal HIE
24 y/male	Porencephaly
26 y/Male	Microdysgenesis

	Disease type

Dentatorubral-pallidoluysian atrophy	
16 y/female	Juvenile
17 y/male	Juvenile
21 y/male	Juvenile
24 y/male	Juvenile
27 y/male	Juvenile
39 y/female	Early adult
40 y/male	Early adult

m: months; y: years; DIC: disseminated intravascular coagulation; HIE: hypoxic ischemic encephalopathy.

**Table 2:** Summary of the subjects in the analysis of cerebrospinal fluid.

Age/sex	Etiology
Control	
1 m/female	Upper respiratory infection
1 m/female	Upper respiratory infection
2 m/male	Viral gastroenteritis
2 m/male	Viral gastroenteritis
6 m/female	Upper respiratory infection
Benign convulsion with mild gastroenteritis	
8 m/male	Viral gastroenteritis
1 y/female	Viral gastroenteritis
2 y/male	Viral gastroenteritis
2 y/male	Viral gastroenteritis
2 y/male	Viral gastroenteritis

	Disease type prognosis

West syndrome	
5 m/female	Cryptogenic
	Normal development
6 m/female	Symptomatic
	Normal development
8 m/female	Cryptogenic
	Delayed development

m: months; y: years.

**Table 3:** Results of the quantitative analysis in the pedunculopontine tegmental nucleus in cases with a history of West syndrome.

	Age	MAP2	AchE	%AchE	TH	%TH	CD	%CD
Controls (*n* = 6)	4 m–38 y	243 (41)	68 (16)	29 (9)	25 (13)	10 (5)	27 (17)	11 (6)
WS/LGS (*n* = 6)	5–18 y	181 (39)	12 (15)	8 (12)	35 (10)	19 (5)	18 (20)	9 (10)
WS (*n* = 6)	5–26 y	184 (38)	29 (39)	13 (18)	27 (18)	16 (10)	31 (14)	17 (8)
Kruskal-Wallis		*P* < 0.05	*P* < 0.05					

“WS/LGS” and “WS” denote cases with a history of West syndrome (WS) and Lennox-Gastaut syndrome (LGS) and cases with a history of only WS, respectively. All results are presented as the mean (SD), and both the total number of neurons that were immunoreactive for each marker and the percentage of neurons immunoreactive for each marker relative to the total neurons are shown. m: months; y: years; MAP2: microtubule-associated protein 2; AchE: acetylcholinesterase; TH: tyrosine hydroxylase; CD: calbindin-D28K.

**Table 4:** Summary of the data in the analysis of the cerebrospinal fluid.

		Acetylcholine	Aspartate	Glutamate	Glycine	GABA	MHPG	HVA	5-HIAA
	Age	(fmol)	(pmol)	(pmol)	(pmol)	(pg/*μ*L)	(pg/*μ*L)	(pg/*μ*L)	(pg/*μ*L)
Controls (*n* = 5)	0–6 m	1.8 (1.2)	305 (179)	94 (39)	477 (214)	44 (13)	46 (33)	204 (133)	912 (520)
West syndrome (*n* = 3)	5–8 m	5.8 (4.3)	81 (25)	37 (7)	137 (49)	22 (7)	77 (24)	203 (64)	698 (225)
CwG (*n* = 5)	8 m–2 y	231 (199)	223 (108)	116 (50)	408 (105)	70 (27)	43 (12)	86 (21)	36 (13)
Kruskal-Wallis		*P* < 0.01			*P* < 0.05	*P* < 0.05			*P* < 0.01

All results are presented as the mean (SD). m: months; y: years; MHPG: 3-methoxy-4-hydroxyphenylethylglycol; HVA: homovanillic acid; 5-HIAA: 5-hydroxyindoleacetic acid; CwG: benign convulsions associated with mild gastroenteritis.
